# Depressive symptoms rather than executive functioning predict group cognitive behavioural therapy outcome in binge eating disorder

**DOI:** 10.1002/erv.2768

**Published:** 2020-07-21

**Authors:** Alexandra E. Dingemans, Gabriëlle E. van Son, Christine B. Vanhaelen, Eric F. van Furth

**Affiliations:** ^1^ Rivierduinen Eating Disorders Ursula Leiden The Netherlands; ^2^ Institute of Psychology Leiden University Leiden The Netherlands; ^3^ Department for Quality of Care GGZ Rivierduinen Leiden The Netherlands; ^4^ Department of Psychiatry Leiden University Medical Center Leiden The Netherlands

**Keywords:** binge eating disorder, depressive symptoms, executive functioning, predictors, treatment outcome

## Abstract

Executive functions play an important role in mediating self‐control and self‐regulation. It has been suggested that the inability to control eating in Binge Eating Disorder (BED) may indicate inefficiencies in executive functioning. This study investigated whether executive functioning predicted cognitive behavioural therapy outcome in BED while accounting for other possible predictors: depressive symptoms, interpersonal factors, eating disorder psychopathology, and self‐esteem. Executive functioning and other predictors were assessed in 91 patients with BED by means of neuropsychological tests and questionnaires at baseline. Eating disorder (ED) symptoms were assessed during treatment at variable time points. Potential predictor variables were investigated using multivariate Cox regression models. Recovery was defined by means of two different indicators based on the Eating Disorder Examination‐Questionnaire: (a) showing a 50% reduction in baseline symptom ED severity and/or reaching the clinical significance cut‐off; and (b) achieving abstinence of objective binge eating. Severity of depressive symptoms was a significant predictor for outcome on both indicators. Patients with no or mild depressive symptoms recovered faster (i.e., 50% reduction in ED symptoms and abstinence of objective binge eating) than those with severe depressive symptoms, which is in line with previous studies. Executive functioning was not related to treatment outcome in this study.

## 1 INTRODUCTION AND AIMS

Binge eating disorder (BED) is characterized by at least weekly recurrent episodes of overeating accompanied by a sense of loss of control without engaging in inappropriate compensatory behaviours (American Psychiatric Association, 2013). According to many international treatment guidelines (Hilbert, Hoek, & Schmidt, 2017) cognitive behavioural therapy (CBT) is the treatment of first choice for BED. However, only half of the patients with BED who complete treatment achieve abstinence of binge eating at post‐treatment and follow‐up (Linardon, 2018). These findings highlight the importance of improving the effectiveness of treatment for BED. Investigating predictors and moderators of treatment outcome is important not only to improve already existing therapies but also to make well‐considered decisions about treatment choices (Dingemans et al., 2016). It may also be speculated that patients with different pre‐treatment characteristics and symptoms do not benefit from the same therapies (Castellini et al., 2012; Penas‐Lledo et al., 2013).

According to the cognitive behavioural model (Fairburn, Cooper, & Shafran, 2003), the key mechanisms that maintain an eating disorder are a low core self‐esteem and overvaluation of weight and shape. It could be hypothesized that there is a smaller likelihood of achieving abstinence of binge eating if these key mechanisms are not successfully reversed in therapy. Furthermore, most models of binge eating (Dingemans, Danner, & Parks, 2017; Fairburn et al., 2003; Heatherton & Baumeister, 1991) incorporate the theory that the emotions of sufferers are poorly regulated and that these individuals often turn to food to escape from, cope with or down‐regulate their emotions (Aldao, Nolen‐Hoeksema, & Schweizer, 2010). According to the escape from awareness model (Heatherton & Baumeister, 1991), binge‐eating episodes are the result of someone's effort to draw attention away from emotional distress and an aversive self‐perception, and towards a narrower focus on the immediate environment (i.e., food).

Executive functions are needed to regulate thoughts, emotions and behaviours. These functions are a set of higher‐order top‐down cognitive processes that are necessary for the cognitive control of behaviour (Lezak, Howieson, & Loring, 2011). Executive functioning is invoked when automatized routines will not work or are not possible (e.g., novel situations; Nigg, 2017). Central to cognitive control of the self is the ability to override or change one's inner responses, as well as to interrupt undesired behavioural tendencies and refrain from acting on them (Tangney, Baumeister, & Boone, 2004). The inability to control eating in BED may therefore imply deficits in executive functioning (Galioto et al., 2012; Manasse et al., 2014). It may be hypothesized that inefficiencies in executive functioning pre‐exist and underlie the development and recurrence binge eating (Lena, Fiocco, & Leyenaar, 2004). A study in healthy adolescents (Lantrip, Isquith, Koven, Welsh, & Roth, 2016) has suggested that those who have less well‐developed executive functions have more difficulty applying the reappraisal strategy and rely more on suppression to regulate their emotions. In other words, those who have better cognitive resources (i.e., executive functions), are able to regulate their emotions in a more healthy way and may be also the ones who benefit more from treatment. CBT requires the patient to carry out homework assignments, restructure thoughts, monitor behaviour and emotions and log progress (Hybel, Mortensen, Lambek, Hojgaard, & Thomsen, 2017). All of these activities require good executive functioning abilities which are therefore a prerequisite for effective treatment (Mohlman & Gorman, 2005). It could be speculated that those individuals who experience more problems in executive functioning are less likely to benefit from CBT (Hybel et al., 2017). Moreover, if patients with poorer executive functioning abilities indeed appear to have a poorer treatment outcome than those with better abilities, extra training of these executive functions could be beneficial for the first group (e.g., cognitive remediation therapy (Danner, Dingemans, & Steinglass, 2015).

The findings of studies in BED on executive functioning however are mixed and the number of studies is limited (Kittel, Brauhardt, & Hilbert, 2015; Lavagnino, Arnone, Cao, Soares, & Selvaraj, 2016; Van den Eynde et al., 2011; Voon, 2015; Wu et al., 2014, 2016; Wu, Hartmann, Skunde, Herzog, & Friederich, 2013). A recent meta‐analysis (Cury et al., 2020) showed alterations in working memory of individuals with BED, relative to obese people without the disorder however the effect size was small. For the other domains (inhibitory control, decision making and cognitive flexibility), no differences in performance were found between the two groups. Due to the limited studies it was not to possible to compare BED with normal weight controls in meta‐analysis.

The aim of the present study was to investigate whether executive functioning abilities predicted treatment outcome in patients with BED, while accounting for other possible predictors that have been found in previous studies: Interpersonal factors (Keel & Brown, 2010), eating disorder psychopathology (Castellini et al., 2012; Grilo, Masheb, & Crosby, 2012; Hilbert et al., 2012), depressive symptoms (Castellini et al., 2012; Grilo et al., 2012) and low self‐esteem (Grilo et al., 2012). The definition of outcome applied here has a considerable influence on outcome estimates (Bjork, Clinton, & Norring, 2011). Applying different outcome criteria provides an opportunity to gain more insight into the process of recovery and the important factors in this process as we have also shown in a previous study (Dingemans et al., 2016). Outcome in this study was defined by means of two different indicators based on the Eating Disorder Examination‐Questionnaire (EDE‐Q): (a) showing a 50% reduction in baseline symptom severity (EDE‐Q global score) or reaching the clinical significance cut‐off (see also Dingemans et al., 2016), and (b) achieving abstinence of objective binge eating. It was hypothesized that patients with BED who experienced difficulties with executive functioning were less likely to have a good outcome of treatment. Those with better executive functioning abilities may be better able to adhere to treatment and thus achieve treatment goals. Furthermore, we hypothesized that the patients with more severe depressive symptoms, more eating disorder psychopathology, lower self‐esteem and more interpersonal distrust would have a worse treatment outcome.

## 2 METHODS

### 2.1 Participants

All consecutive patients who presented themselves for treatment at our specialized eating disorder treatment centre between October 2012 and February 2016 with a primary diagnosis of BED according to DSM‐5 criteria (American Psychiatric Association, 2013) were asked to participate in this study (see for more details (Dingemans, Vanhaelen, Aardoom, & van Furth, 2019). Ninety‐five percent of the patients starting treatment agreed to participate in the study. During the intake procedure diagnoses were made by a multidisciplinary team of experienced psychologists and psychiatrists specialized in eating disorders. The questions asked in the intake were taken from two standardized semi‐structured interviews: the Eating Disorder Examination (Fairburn & Cooper, 1993) and the Longitudinal Interval Follow‐up Evaluation (Keller et al., 1987; Krämer, 1996).

### 2.2 Procedure and design

An information letter about the study was sent to the eligible participants prior to the start of treatment. Subsequently, they were provided with written and verbal information in person by one of the researchers. Those who were willing to participate, were asked to sign an informed consent form. The study assessments were conducted prior to the start of treatment as part of the Routine Outcome Monitoring. The duration of the first baseline assessment was approximately one and a half hours, in which five questionnaires and six neuropsychological tests (see Measures) were completed. The researchers were trained to administer the neuropsychological tests. None of them had a therapeutic relationship with any of the participants. The EDE‐Q was completed during the follow‐up assessments (every 6 months). ROM continues for as long as the patient is being treated. Therefore the total number of assessments per patient varies as it depends on the duration of treatment.

The study was approved by the local ethics committee (METIGG).

### 2.3 Treatment

The participants started in an open group with a maximum of six sessions. The topics of the sessions included the formulation of individual treatment goals, psycho‐education (psychologist, dietician and physical therapist), engaging family and/or friends, and enhancing the motivation to change. Subsequently, CBT was conducted on an outpatient basis in a closed group format with a maximum of 10 participants. The treatment consisted of 20 group sessions conducted over a 30‐week period. The first 10 sessions were once a week and the final 10 sessions were every 2 weeks. Each session lasted 2 hr. Two therapists (psychologist and psychiatric nurse) trained in CBT conducted 16 sessions and a dietician four sessions. The CBT sessions were semi‐structured and problem‐oriented. The main focus of the sessions was on the patients' present and future rather than their past. The CBT for BED group consisted of three phases. In the first phase (Sessions 1–10), the main goal was to develop a regular eating pattern and to resist the urge to binge eat. They were asked to keep a daily food diary. Furthermore, they learned to identify and correct dysfunctional cognitions and avoidance behaviours related to eating. Replacing these behaviours with healthier, self‐enhancing responses was another goal. In the second phase (Sessions 11–18) underlying problems such as body image, self‐esteem, stress management, problem solving, assertiveness and weight loss issues were addressed. The third and final phase of the treatment (Sessions 19 and 20) patients were asked to make their own relapse prevention plan. Homework assignments were part of all sessions, with feedback being given on the food diaries and homework assignments. An earlier version of this treatment manual was used in a previous study by our group (Dingemans, Spinhoven, & van Furth, 2007). If necessary, treatment could be prolonged with other follow‐up treatment such as relapse prevention, weight loss management or schema therapy.

## 3 MEASURES

### 3.1 Demographic and clinical variables

Participants' marital status, socio‐economic status (homemaker/retired, full‐time job/student, part‐time job, or disabled), educational level (low, medium, high), age, duration of illness, use of psychotropic medication (yes/no) were recorded.

### 3.2 Eating disorder psychopathology

The EDE‐Q (Fairburn & Cooper, 1993) is a 36‐item self‐report questionnaire used to assess eating disorder‐specific psychopathology over the previous 28 days. Twenty‐two items assess the core attitudinal features of eating disorder pathology and were answered on a seven‐point Likert scale. A global score was calculated by summing up and averaging the 22 items. A higher score reflects more psychopathology (range 0–6). Fourteen items assess the frequency of core ED behaviours, including frequency of objective and subjective binge eating episodes. Weight and height were measured and BMI was calculated accordingly (weight/height^2^). EDE‐Q global score, frequency of objective binge eating episodes and BMI were used as predictors in the present study. Several studies provided support for the reliability and validity of the EDE‐Q for assessing eating disorder symptoms (Aardoom, Dingemans, Slof‐Op't Landt, & Furth, 2012; Berg, Peterson, Frazier, & Crow, 2012). The EDE‐Q global score was found to be highly accurate at discriminating individuals with an eating disorder from those without (Aardoom et al., 2012).

### 3.3 Interpersonal distrust

The Eating Disorder Inventory II (EDI‐II)(van Strien, 2002) is a self‐report questionnaire measuring specific ED‐related psychological and behavioural characteristics. In this study the interpersonal distrust subscale is used since this appeared to be a predictor for treatment outcome in a previous study (Dingemans et al., 2016). Higher scores indicated greater levels of pathology The test–retest reliability for the EDI‐II subscale was relatively high, indicating a good and acceptable stability over time (*r* = 0.86; Thiel & Paul, 2006). The internal consistency of the subscale in the present study appeared to be good (Cronbach's alpha 0.83). The internal consistency of the subscale in this study appeared to be good (Cronbach's alpha 0.83).

### 3.4 Depressive symptoms

The Dutch version of the BDI‐II (Beck, Steer, & Garbin, 1988; Van der Does, 2002) is a 21 item (scored from 0 to 3) questionnaire which measures the severity of depressive. The total score is the sum of the 21 items (range 0–63). The following cut‐off scores were used: no or minimal depression is <13; mild depression is 14–19; moderate to severe depression is 20–28; and severe depression is 29–63. The internal consistency and test–retest reliability of this version was high for a psychiatric outpatient group and a healthy control group (Van der Does, 2002).

### 3.5 Self‐esteem

Rosenberg's Self‐Esteem scale (RSE; Rosenberg, 1965) consists of 10 questions on self‐worth. Scores were summed and could range from 10 to 40, with higher scores reflecting higher self‐esteem.

The RSE scale has a high construct validity. The mean construct validity across 53 nations was substantial (Schmitt & Allik, 2005). The internal consistency of the RSE in the present study is high (Cronbach's alpha = 0.93).

### 3.6 Response inhibition

To measure response inhibition the Stroop Test was used (Stroop, 1935) which consisted of three trials. The first trail was a list of the following colours on paper: red, green, blue and yellow. The patients were asked to read the words aloud. The second trail consisted of small rectangles in the same colours on paper. The patients were asked to name the colours of the rectangles. In third and final trail, individuals were given a list of names of the four colours, but written in a different colour text than that which they actually represent, and they were asked to read the words aloud. Time to complete was measured with a stopwatch. The measure for response inhibition in this study was the relative difference in time between trial 1 and 3. A smaller difference reflects better response inhibition.

### 3.7 Decision‐making

Decision‐making ability was measured by means of a computerized adaptation (Mueller, 2009) of the Iowa Gambling Task (IGT; Bechara, Damasio, Damasio, & Anderson, 1994). The purpose of this task is to test patients' ability to resist immediate gains in favour of a longer‐term positive outcome. At the start of the task each participant was given a virtual amount of 2,000 dollars of play money. They were instructed to make as much profit as possible. Participants were presented with four virtual decks of cards (A‐B‐C‐D) on a computer screen. When they selected a card of one of the decks they either (a) gained, or (b) gained and lost, virtual money. Decks A and B were disadvantageous in the long run because the total gains were lower than the total losses. Decks C and D were advantageous because, although the gains were lower, the losses were also lower. Decision‐making ability was measured by calculating a total score (the total number of advantageous choices (decks C and D) minus the number of disadvantageous choices (decks A and B). Higher scores were indicative for a better performance.

### 3.8 Set‐shifting

A computerized adaptation of the Trail Making Test (TMT; Reitan, 1956) was used (Buro Tester, 2006b) to measure set‐shifting ability. In each of the two trials 25 dots were presented on a computer screen. First, patients were instructed to complete a 25‐item numeric sequence (Trail A: 1–2‐3, etc.) and then a 25‐item numeric‐alphabetical sequence (Trail B: 1‐A‐2‐B‐3‐C, etc.) as quickly as possible. In order to control for baseline motor speed, set‐shifting outcome score was defined as the relative difference in time (seconds) between part B and part A. A higher score reflected worse set‐shifting abilities.

A computerized adaptation (Buro Tester, 2006a) of the Wisconsin Card Sorting Task (WCST; Heaton et al., 1993) was the second task in this study to measure set‐shifting ability. Patients were instructed to match stimulus cards with one of four category cards that vary in geometric shape, color, and number of items per card. In advance, the participant had no knowledge of the sorting rule (i.e., shape, colour or number) required for correct matching. After each guess, the words ‘right’ or ‘wrong’ appeared on the screen. The participant therefore had to discover the sorting rule, which changed after 10 consecutive correctly matched cards without warning during the course of the task. The number of perseverative errors was used as a measure of set‐shifting ability.

### 3.9 Working memory

Working memory was assessed by the subscale Digit Span of the Wechsler Adult Intelligence Scale (WAIS‐IV; Wechsler, 2012). First, the experimenter read aloud sets of digits which the patient had to repeat forwards. Second, other sets of digits are read aloud but now the patient had to repeat them backwards. After two incorrect answers of the same number of digits, the test was ended. In this study the digit span backwards is used as a measure for working memory. A better attention and working memory function was reflected by a higher score on the digit span backwards.

### 3.10 Central coherence

The Rey Complex Figure Test (RCFT; Osterrieth, 1944) is one of the most widely used neuropsychological test to assess visual spatial coherence. Participants were asked to copy the complex geometric figure. The traditional task also involves asking participants to recall the figure without prior warning after a time interval. When assessing central coherence abilities however, it is the copy aspect of the task which is of interest. In accordance with Booth's scoring system (Booth, 2006), an order of construction score (i.e., the order in which the elements of the figure were drawn) and style score (i.e., continuity of drawing) were calculated. The Central Coherence Index (CCI) is the mean score of the order of construction and the style score. A lower CCI reflects weaker central coherence.

### 3.11 Executive functioning in daily life

The Behavior Rating Inventory of Executive Function Adult version (BRIEF‐A‐SR) (Roth, Isquith, & Gioia, 2004) is a 75‐item questionnaire which assesses a person's own perception of their difficulties in executive functioning in daily life (Noens and Scholte, 2011). Two summary index scales were calculated, that is, the behavioural regulation index (including the subscales inhibit, shift, emotional control, and self‐monitor) and the metacognition index (including the subscales initiate, working memory, plan/organize, task monitor, and organization of materials). The scores were converted into T‐scores, with a mean of 50 and a standard deviation of 10 based on age (Noens & Scholte, 2011). The questionnaire has been found to adequately assess the impact of executive functioning difficulties in everyday tasks and common situations. The BRIEF‐A demonstrated to have a high internal consistency reliability and good ecological validity (Rouel, Raman, Hay, & Smith, 2016). Higher scores reflect more problems in executive functioning in daily life.

### 3.12 Statistical analyses

The two outcome variables in this study to measure treatment response were based on the EDE‐Q: (a) showing a 50% reduction in baseline symptom severity (EDE‐Q global score) or reaching the clinical significance cut‐off, and (b) achieving abstinence of objective binge eating. The clinical significance cut‐off point was calculated according to the ‘C’ method described by Jacobson and Truax (1991), namely: ((M_clinicalgroup_ × SD_normalgroup_) + (M_normalgroup_ × SD_clinicalgroup_))/(SD_normalgroup_ + SD_clinicalgroup_). All psychometric data used in this formula were taken from the study by Aardoom et al. (2012). The calculated clinical cut‐off point was 2.17 (for more details see Dingemans et al., 2016).

Survival analyses (Cox regression) were used to measure the association between possible predictor variables and the outcome variable. When follow‐up measurements were discontinued, the survival time was censored at the last point at which a ROM assessment was completed when a response (the event under study) was not achieved (Dingemans et al., 2016). Survival time was defined as the number of weeks since baseline (T0). Cases were excluded from the analyses if: (a) there were fewer than two assessments; (b) the EDE‐Q global score was missing at the second assessment; or (c) the EDE‐Q global score was below 0.5 at baseline (in line with (van Noorden et al., 2012) and (Dingemans et al., 2016)).

The first step was to investigate the association between possible predictors (see Table 2) and treatment response. When correlations were found to be significant and above the value of *r* = .68 (multicollinearity) (Taylor, 1990), one or more of these predictor variables were excluded from the analysis. Subsequently, potential predictor variables were investigated in univariate Cox regression models, and corresponding univariate hazard ratios (recovery/response) were computed. Then, continuous variables were redefined as categorical variables to allow presentation in Kaplan–Meier curves. In order to facilitate the comparability of effect sizes between the predictors, the predictor variables were standardized by calculating *z*‐scores.

The second step was to include predictor variables in the multivariable Cox regression model if the Hazard Ratios had a *p*‐value lower than .10 (Field, 2013). Backward multiple regression analyses were conducted using a conditional parameter criterion (alpha *p* < .05). Predictors were found to be significant at an alpha level of *p =* .05. Since this is a naturalistic study with no randomization, the variables age, degree of comorbidity and education level could be confounders (Dingemans et al., 2016), therefore these variables are entered in the second step in the multiple regression analyses. The software used was SPSS version 22.0 (IBM Statistics SPSS).

## 4 RESULTS

### 4.1 Participants

Ninety‐one patients with BED were assessed at baseline, of whom 85 (93%) had two or more follow‐up assessments and were included in the present study. The mean age of the sample at baseline was 33.8 years (*SD* = 9.5; range 20–60). The mean duration of the eating disorder was 15.9 years (*SD* = 11.3). Approximately one third of the patients (37%, see Table 1) used psychotropic mediation of which selective serotonin reuptake inhibitors (SSRI's) were the most prescribed. See Table 1 for more demographic and clinical characteristics.

**TABLE 1:** Demographic and clinical characteristics of patients with binge eating disorder (BED; *N* = 91)

	*M* (SD) or *n* (%)	Range
Age (years), *M* (*SD*)	33.8 (9.5)	20–60
Objective binge eating episodes (28 days), *M* (*SD*)	12.2 (9.8)	1–50
EDE‐Q global score, *M* (*SD*)	3.8 (0.9)	0.9–5.8
BMI (kg/m^2^), *M* (*SD*)	37.9 (6.5)	24.2–53.4
BDI‐II total score, *M* (*SD*)	26.5 (11.3)	4–54
Duration of illness (years), *M* (*SD*)	15.9 (11.3)	1–44
Psychotropic medication (present: *n*, %)		
No	56 (62%)	
Yes	34 (37%)	
SSRI	21	
TCA	2	
Methylphenidate	2	
Benzodiazepine	3	
More than one (including antipsychotic and/or epileptic medication)	6	
Missing	1 (1%)	
Comorbid psychiatric disorders (present: n, %)		
None	51 (56%)	
One	31 (34%)	
Two or more	8 (8%)	
Missing	1 (1%)	
Gender (*n*, %)		
Female	85 (93%)	
Male	6 (7%)	
Living situation, *n* (%)		
Living alone	44 (48%)	
Living together/with children	39 (43%)	
Other	8 (9%)	
Socio‐economic status, *n* (%)		
School/study	11 (12%)	
Employed	49 (54%)	
Unemployed/homemaker	11 (12%)	
Sick leave/disabled	18 (20%)	
Other	2 (2%)	
Highest educational level, *n* (%)		
Lower secondary school	9 (10%)	
Higher secondary school	43 (54%)	
Bachelor/master	39 (36%)	
Response inhibition		
Stroop test (trial 3/trial 1), *M* (*SD*)	1.65 (0.34)	1.09–3.58
Decision making		
Iowa gambling task total score, *M* (*SD*)	8.9 (22.82)	−70 to 76
(Advantage minus disadvantage decisions)		
Set‐shifting		
Trail making test (trail C/trail A), *M* (*SD*)	1.47 (0.44)	0.77–2.86
WCST number of perseverative errors, *M* (*SD*)	17.83 (8.47)	0–49
Working memory		
Digit span backward, number correct, *M* (*SD*)	6.90 (2.11)	2–12
Central coherence		
Rey complex figure test, CCI, *M* (*SD*)	1.85 (0.4)	0.58–2.75
Executive functioning in daily life		
BRIEF‐A behavioral index, *M* (*SD*)	64.05 (10.40)	40–86
BRIEF‐A metacognition index, *M* (*SD*)	67.30 (11.10)	39–92

Abbreviations: BDI‐II, beck depression inventory‐II; BMI, body mass index; BRIEF‐A, behaviour rating inventory of executive function‐adult version; CCI, central coherence index; DE‐Q, eating disorder examination questionnaire; SSRI, selective serotonin reuptake inhibitor; TCA, tri‐cyclic anti‐depressant; WCST, Wisconsin card sorting task.

#### 4.1.1 Univariate analyses

None of the potential predictors appeared to have a strong correlation (*r* > .68; Taylor, 1990) with each other and with the dependent variable (EDE‐Q total score). Only the two variables age and duration of illness appeared to be strongly associated (*r* = 0.69). Age was used as a predictor in the analyses. Compared to duration of illness, age is a variable that can be established with higher reliability.

Criterion 1: 50% reduction of baseline EDE‐Q score and/or clinical cut‐off score.

The overall median survival time of this combined criterion was 75 weeks (95% CI: 41–109). Of the 19 predictors, only three significantly predicted a good outcome (see Table 2): patients with no to mild depressive symptoms, those with better self‐esteem and those who did not use psychotropic medication were more likely to have a 50% reduction of baseline eating disorder psychopathology (or have a score below the clinical cut‐off) than those with more severe depressive symptoms and worse self‐esteem and with medication. Furthermore, speed of recovery was faster in the first groups than in the latter ones.

**TABLE 2:** Univariate hazard ratios of treatment outcomes in a large naturalistic cohort of patients with an eating disorder

Predictor variables		EDEQ 50% and/or below clinical cut‐off	Abstinence objective binge eating
		Hazard ratio (95% CI)	Hazard ratio (95% CI)
Age (years)		1.06 (0.79–1.43)	1.04 (0.81–1.34)
Depressive symptoms (BDI‐II)		0.62 (0.45–0.87)***	0.73 (0.55–0.95)**
ED psychopathology	EDE‐Q binge eating episodes/28 days	0.85 (0.61–1.19)	n.a.
	EDE‐Q total score	n.a.	0.76 (0.59–0.99)**
Interpersonal distrust (EDI‐II)		0.78 (0.58–1.04)*	1.23 (0.93–1.62)
Self‐esteem (RSE)		1.43 (1.07–1.91)**	1.26 (1.00–1.59)**
Comorbid axis I disorder	No (0) Yes (1)	0.95 (0.51–1.77)	0.86 (0.51–1.44)
Sex	Female (0) Male (1)	0.67 (0.20–2.18)	0.65 (0.23–1.80)
Medication	No (0) Yes (1)	0.36 (0.18–0.74)***	0.58 (0.34–1.00)**
Highest educational level	low	1.03 (0.34–3.09)	2.00 (0.84–4.76)
	Intermediate	0.64 (0.49–1.80)	1.47 (0.85–2.56)
	High	1.00	1.00
BMI		1.12 (0.83–1.51)	0.90 (0.71–1.15)
Response inhibition	Stroop test (trial 3/trial 1)	0.72 (0.49–1.08)	0.97 (0.76–1.23)
Decision‐making	Iowa gambling task total score	0.79 (0.54–1.16)	0.90 (0.68–1.19)
Set‐shifting	Trail making test (trail C/trail A)	0.99 (0.72–1.39)	1.05 (0.81–1.36)
	WCST number of perseverative errors	1.20 (0.88–1.64)	1.15 (0.91–1.46)
Working memory	Digit span backwards, number correct	0.91 (0.68–1.21)	0.91 (0.71–1.16)
Central coherence	Rey complex figure test, CCI, *M* (*SD*)	1.26 (0.63–2.53)	1.22 (0.75–1.99)
Executive functioning daily life	BRIEF‐A behavioural index	0.78 (0.58–1.05)*	0.81 (0.63–1.04)
	BRIEF‐A metacognition index	0.78 (0.57–1.07)	0.82 (0.63–1.06)

Abbreviations: BDI‐II, beck depression inventory—version II; BRIEF‐A, behaviour rating inventory of executive function‐adult; CCI, central coherence index; CI, confidence interval; EDE‐Q, eating disorder examination questionnaire; EDI‐II, eating disorder inventory II; RSE, Rosenberg self‐esteem; WCST, Wisconsin card sorting test.

* *p* ≤ .10.

** *p* ≤ .05.

*** *p* < .001.

Criterion 2: abstinence of objective binge eating.

The overall median survival time of this combined criterion was 48 weeks (95% CI: 43–53). Of the 19 predictors, four significantly predicted outcome. Less severe depressive symptoms and eating disorder psychopathology, more self‐esteem, and no psychotropic medication use at baseline predicted a good outcome faster i.e., abstinence of objective binge eating).

#### 4.1.2 Multivariate analyses

**FIGURE 1:**
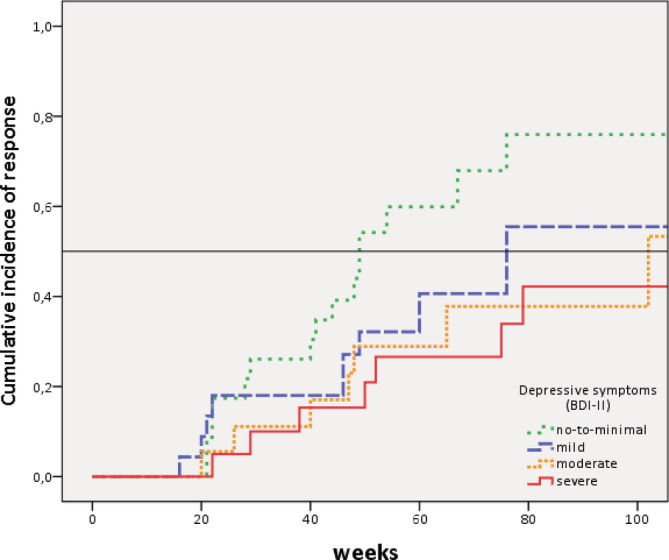
Kaplan–Meier curves for good outcome (50% reduction EDE‐Q score or below clinical cut‐off) according to depressive symptoms (BDI‐II) [Colour figure can be viewed at wileyonlinelibrary.com]

Starting with the 50% reduction of baseline EDE‐Q score and/or clinical cut‐off score (Criterion 1), the three significant predictors plus the two trend significant predictors (interpersonal distrust (EDI‐II) and the BRIEF‐A BRI) found in the univariate analyses were entered into the multivariate cox regression analysis (backwards). Depressive symptoms (as measured by the BDI‐II) and psychotropic medication use (yes/no) at baseline appeared to be the only predictors for a 50% reduction in eating disorder psychopathology or clinical cut‐off. Higher baseline BDI‐II scores were associated with a decrease in the likelihood of a good outcome (HR = 0.70 [95% CI: 0.50–0.97], *p* = .03; see Figure 1) and using medication decreased the chance of good outcome (HR = 0.42 [95% CI: 0.20–0.87], *p* = .02). Those patients who used medication were more likely to have a comorbid DSM diagnosis (*χ*[2] = 15.1, *p* < .01). In a second step age, comorbidity yes/no) and educational level were entered as possible confounders. The results did not change: Depressive symptoms and psychotropic medication use were still significant predictors (HR = 0.63 (95% CI: 0.44—0.88), *p* = .008 and HR = 0.33 (95% CI: 0.15—0.72), *p* = 0.005, respectively).

With regard to the second criterion, only one of the four predictors from the univariate analyses was found to predict a better outcome in the multivariate analyses. Those patients with no or mild depressive symptoms were more likely to achieve abstinence of objective binge eating than those with more severe depressive symptoms (HR 0.74 [CI: 0.74–0.97], *p =* .03). Also, after entering the three above mentioned possible confounders, the results did not change (depressive symptoms: HR = 0.68 [95% CI: 0.51–0.91], *p* = .008).

## 5 DISCUSSION

The aim of the present study was to investigate whether executive functioning abilities predicted treatment outcome in patients with BED, while accounting for other possible predictors that have been found in previous studies: Interpersonal factors (Dingemans et al., 2016; Keel & Brown, 2010), eating disorder psychopathology (Castellini et al., 2012; Grilo et al., 2012; Hilbert et al., 2012), depressive symptoms (Castellini et al., 2012; Grilo et al., 2012) and low self‐esteem (Dingemans et al., 2016; Grilo et al., 2012). As expected the median survival time for abstinence of binge eating was much shorter than the median survival time of achieving a 50% reduction of eating disorder cognitions and attitudes (or reaching an EDE‐Q global score that fell within the healthy range), which is in line with many other studies that found that psychological recovery follows behavioural recovery (e.g., (Bardone‐Cone et al., 2010; Hilbert et al., 2012).

Contrary to our expectations, baseline executive functioning abilities did not predict the speed of outcome (i.e., 50% reduction of eating disorder psychopathology as well as abstinence of binge eating) of treatment in patients with BED. These results suggest that inefficiencies in executive functioning are only weakly related or not at all related to becoming abstinent of binge eating or to reduce eating disorder psychopathology. An explanation may be that the assessment of neuropsychological tests is carried out in standardized conditions structured by the assessors (Blijd‐Hoogewys, Bezemer, & van Geert, 2014) and that performances on these tests are just snapshots and may therefore not be representative of the more complex functioning in daily life. The traditional neuropsychological tests may not be sensitive enough in detecting inefficiencies in executive functioning. Moreover, the tests were developed with the intent of assessing brain trauma and lesions, not psychiatric populations (Dahlgren, Hage, Wonderlich, & Stedal, 2019). However, also the baseline indexes of BRIEF‐A which measure executive functioning in daily life and are supposed to be more sensitive in detecting different levels of executive functioning (Rouel et al., 2016), failed to predict the speed of outcome in patients with BED. Third, our previous study (Dingemans et al., 2019) demonstrated that only a minority of patients with BED had significant deficits in executive functions compared to healthy controls, which could also explain why executive functioning was not a predictor of outcome. A fourth and last explanation may be that some of the aspects of executive functioning are implicitly addressed in CBT, like planning, response inhibition and set‐shifting. One could argue that those with poor executive functioning benefit from CBT because they do learn to improve these abilities.

In line with our hypothesis, patients with no or minimal depressive symptoms at baseline appeared to have recovered faster than those with more severe depressive symptoms. This is also in line with previous studies (see for review (Dingemans et al., 2017) which suggested an association between depressive symptoms, acute sad mood, and binge‐eating behaviour, and indicated that higher levels of depression are related to more severe binge eating. Most theoretical models of eating disorders (Fairburn et al., 2003; Heatherton & Baumeister, 1991; Polivy & Herman, 1985) argue that negative mood is involved in the maintenance of eating disorders in general. In binge eating disorder, poor mood appears to precede binge‐eating episodes and binge eating may be an attempt to down‐regulate and suppress this emotional distress (Dingemans et al., 2017). Individuals with BED appear to lack effective and healthy emotion regulation strategies. They have a tendency to avoid (Rosenbaum & White, 2016) and suppress their unwanted emotions or engage in rumination (Prefit, Candea, & Szentagotai‐Tatar, 2019). They suppress their emotions by means of eating large quantities of food while being out of control. Moreover, in contrast with healthy controls they appear to use adaptive strategies, such as reappraisal, far less. Poor regulation of negative emotions may therefore be an underlying process that is involved in the maintenance of BED (Dingemans et al., 2017). Also, ecological momentary assessment (EMA) research has highlighted that momentary relationships between affect and binge eating exist (Engel et al., 2016), however it is yet unclear how, when, and for whom these relationships emerge in individuals with BED (Smith et al., 2020). Additional treatment could be offered for the depressive symptoms to enhance the effectiveness of CBT for example by means of anti‐depressant medication or therapy directed at improvement of affect regulation skills.

An unexpected predictor for poor outcome appeared to be the use of psychotropic medication. Those who used medication were less likely to have a quick reduction of the baseline severity of eating disorder psychopathology than those who did not. Those patients who had comorbid psychiatric disorders were more likely to use medication. The fact that having a comorbid disorder appeared not to be a significant predictor in the present analyses may be explained by the fact that the presence of comorbid disorders may be underreported during intake. Maybe use of psychotropic medication at intake may be a better proxy for having more comorbid psychiatric disorders. Other explanations may be that the current medication is no longer effective and that medication use is a confounder in the relationship between comorbidity and outcome. Ineffective medication may be a marker for an untreated comorbid disorder. The clinical implications of this study are that therapists should pay close attention to patients with BED who display depressive symptoms and those who already use medication at the beginning of treatment. An appointment with the psychiatrist at the beginning of treatment is recommended.

A strength of the present study is the representable cohort of patients with BED seeking help in a specialized centre for eating disorders. All consecutive patients were asked to participate in the study and only a few patients declined. Furthermore, we used neuropsychological tests as well as a questionnaire to assess executive functioning. This study should also be considered in the light of a few limitations. First, as discussed above, comorbid disorders are probably underreported. Second, our sample size is relatively small. Furthermore, our selection of neuropsychological tests may be debated. Although we used an extensive battery of widely used neuropsychological tests, other tests could have been chosen. Finally, interpersonal factors were only measured by a subscale of the EDI‐II (van Strien, 2002). Since interpersonal factors are one of the known risk factors for the development of BED a more extensive questionnaire like the inventory of interpersonal problems (Horowitz, Rosenberg, Baer, Ureno, & Villasenor, 1988), should be considered. In conclusion, the present study showed that speed of recovery was predicted by the severity of the depressive symptoms, which is in line with previous studies on this subject. Contrary to expectations, baseline executive functioning was not related to speed of recovery.

## CONFLICT OF INTEREST

All authors declare that they have no conflict of interest.

## AUTHOR CONTRIBUTIONS

Alexandra E. Dingemans, Gabriëlle E. van Son, Christine B. Vanhaelen and Eric F. van Furth made substantial contributions to the design, or acquisition of the data, or to the analysis and interpretation of the data. Alexandra E. Dingemans and Gabriëlle E. van Son were involved in drafting the manuscript or revising it critically for important intellectual content. Alexandra E. Dingemans, Gabriëlle E. van Son, Christine B. Vanhaelen and Eric F. van Furth gave final approval of the version to be published. Alexandra E. Dingemans, Gabriëlle E. van Son, Christine B. Vanhaelen and Eric F. van Furth agreed to be accountable for all aspects of the work.
